# Experiences of homosexual patients’ access to primary health care services in Umlazi, KwaZulu-Natal

**DOI:** 10.4102/curationis.v38i2.1522

**Published:** 2015-09-28

**Authors:** Nokulunga H. Cele, Maureen N. Sibiya, Dudu G. Sokhela

**Affiliations:** 1 Department of Health, University of KwaZulu-Natal, South Africa; 2 Department of Nursing, Durban University of Technology, South Africa

## Abstract

**Background:**

Homosexual patients are affected by social factors in their environment, and as a result may not have easy access to existing health care services. Prejudice against homosexuality and homosexual patients remains a barrier to them seeking appropriate health care. The concern is that lesbians and gays might delay or avoid seeking health care when they need it because of past discrimination or perceived homophobia within the health care thereby putting their health at risk.

**Aim of the study:**

The aim of the study was to explore and describe the experiences of homosexual patients utilising primary health care (PHC) services in Umlazi in the province of KwaZulu-Natal (KZN).

**Method:**

A qualitative, exploratory, descriptive study was conducted which was contextual in nature. Semi-structured interviews were conducted with 12 participants. The findings of this study were analysed using content analysis.

**Results:**

Two major themes emerged from the data analysis, namely, prejudice against homosexual patients by health care providers and other patients at the primary health care facilities, and, homophobic behaviour from primary health care personnel.

**Conclusion:**

Participants experienced prejudice and homophobic behaviour in the course of utilising PHC clinics in Umlazi, which created a barrier to their utilisation of health services located there. Nursing education institutions, in collaboration with the National Department of Health, should introduce homosexuality and anti-homophobia education programmes during the pre-service and in-service education period. Such programmes will help to familiarise health care providers with the health care needs of homosexual patients and may decrease homophobic attitudes.

## Introduction

Access to health care is a fundamental principle of health care systems around the world and is a fundamental human right of all citizens irrespective of race, class, religion, gender or sexual orientation. Access to health care can be defined as the ability of an individual to identify their health needs and to seek health care services and obtain the relevant care for their health needs (Levesque, Harris & Russell 2013:1). The Constitution of the Republic of South Africa, Section 27(1)(a), states that all citizens have the right to access health care services (Republic of South Africa 1996:6–8). The priority for public health is to eliminate health disparities in all spheres together with adverse health outcomes by improving access and utilisation of health care services (Graves 2009:46). Despite the recognition and increased awareness of homosexuality and the health needs of homosexual patients, negative attitudes persist and affect the way in which they can access health care services (Hayman *et al.* 2013:121).

A 2009 survey conducted amongst South African students in the Western Cape regarding their attitudes and beliefs towards homosexuality, found that 44% of them disapproved of the idea of homosexuality being acceptable in South Africa, and 41% believed that granting equal rights to homosexual individuals by the constitution was correct and acceptable (Mwaba 2009:803). The results of this survey indicate the general attitude some members of the community have towards homosexual patients and homosexuality. Homophobia, which is ‘the irrational fear and hatred of people who are attracted to the same sex’ (Müller 2013:2), is most threatening for lesbians. Many South African lesbians are living in fear of being ‘found out’ and becoming victims of discrimination and possible physical harm, including ‘corrective rape’ (Ochse 2011:3–4). Such sexual violence, driven by homophobia, places lesbians at high risk of HIV infection (Müller 2013:2).

Men who have sex with men (MSM) reported having a fear of being judged by health care providers and getting treated badly as a result of disclosure (Rispel *et al.* 2011:47). Other studies (Kennedy *et al.* 2013:3; Rosenstreich, Comfort & Martin 2011:2) assert that homosexual patients experience stigmatisation when accessing health services. O’ Byrne and Watts (2014:21) point out that many homosexual patients avoid doing an HIV test because of hesitance and fear of stigmatisation. Some people in society believe that homosexual patients have violated cultural gender norms, and that stigma also adds barriers to access health care settings (Rounds, McGrath & Walsh 2013:99).

Many studies (for example Banwari *et al.* 2015; O’ Byrne & Watts 2014; Ochse 2011; Rispel *et al.* 2011) reveal that lesbians (females) and gays (males), commonly known as homosexuals, experience challenges regarding access to health care services, and this has become a serious concern. The concern is that lesbians and gays might delay or avoid seeking health care when they need it because of past discrimination or perceived homophobia within the health care, thereby putting their health at risk (Müller 2013:2). For example, Bjorkman and Malterud (2009:239) found that lesbians were not following the sequence of screening programmes for females, such as pap smears. They emphasised that lesbians might suffer from health problems to a greater extent than heterosexuals or the general female population as a result of marginalisation.

Rispel *et al.* (2011:138) conducted a survey in South Africa regarding perceptions, preferences and utilisation of health care services by homosexual patients. They found that negative attitudes from health care providers discouraged them from disclosing their sexual orientation, often resulting in inadequate treatment and unmet health care needs.

Homosexual patients are affected by a range of social and structural factors around their environment, and as a result have unique health needs that might not be met by existing health care services because of ignorance by health care providers but also fear of disclosure by homosexual patients (Mitra & Globerman 2014).

In their study, Rounds *et al.* (2013:99) found that homosexual patients experience verbal abuse as well as many other forms of discrimination and a general lack of satisfactory care, including from health care professionals refusing to provide them with treatment. Eaton *et al.* (2015) report on a study in which homosexual men state that the health services offered to them were substandard. Rispel *et al.* (2011:147–149), in their study of South African homosexual men, found that some participants perceived the private health care sector to be more understanding than the public health sector.

## Profile of the researcher

The principal investigator was employed as a Clinical Nurse Specialist in one of the PHC (primary health care) facilities at Umlazi township in KZN at the time of conducting this study. She noticed that very few homosexual patients visited the clinic and that the majority of them only reported to the clinic during late stages of their illnesses or when they had complications.

## Problem statement

The rights of all South African citizens are enshrined in the Constitution of the Republic of South Africa which ensures non-discrimination by sexual orientation (Republic of South Africa 1996:13), yet it seems that homosexual patients are still discriminated against when seeking health care. They are not yet free in South African society as a result of the challenges that they daily encounter such as social stigma and homophobic attacks (Ochse 2011:4), and are often fearful and hesitant to access health care services owing to the possibility of discrimination by health care providers.

## Aim of the study

The aim of the study was to explore and describe the experiences of homosexual patients utilising PHC services in Umlazi in KZN.

## Research method design

### Design

A qualitative research study that was exploratory, descriptive and contextual in nature was conducted. A qualitative study has a subjective approach and is used to define life experiences and give them meaning (Grove, Burns & Gray 2013:23). The study provides an overview of the real life situation of homosexual patients, particularly lesbians, regarding access to health care services at the PHC level in Umlazi.

The exploratory design explores the dimensions of a phenomenon in order to better understand the context in which an intervention unfolds. The exploratory design sheds light on the various ways in which a phenomenon is manifested and on underlying processes (Polit & Beck 2012:640). The researcher used an exploratory design to better understand the accessibility of PHC services for homosexual patients.

A descriptive research design provides an accurate account of the characteristics of a particular group in real-life situations for the purposes of describing what exists, determining the frequency with which something occurs, and categorising information (Grove *et al.* 2013:26). The study was contextual in nature as the validity of the findings is only claimed in a specific context. This design was used to obtain more information about characteristics of homosexual patients regarding their experiences of utilising primary health care services in Umlazi. The purpose of a descriptive study is to provide a picture of what is happening in real life situations (Grove *et al.* 2013:215).

### Sampling and sampling technique

Purposive sampling was used to select the study participants. In qualitative research, purposive sampling is used to recruit participants for whom the research topic is relevant and the goal is to gather information about their experiences (Knudsen *et al.* 2012:85). Purposive sampling was used because it enabled the researcher to select informative cases that could provide rich and substantial information based on the personal experiences of homosexual patients regarding accessing and using PHC services in the Umlazi area. Inclusion criteria were 18 years of age or older, self-identified as either lesbian or gay, experience of PHC services in Umlazi within the past year, and able to conduct a conversation in either English or isiZulu.

Purposive sampling was modified by time-space probability based sampling. This technique is used when there is a relatively small number of people who usually gather at a specific place and time and who experience stigma and discrimination. These samples are often called hard-to-reach populations and include homosexual patients (Berry *et al.* 2013:39; Semaan 2010:61). Potential participants were approached at a gay club with the permission of the club owners. The recruitment flyers were posted at the clubhouse one week before the researcher began data collection.

According to Polit and Beck (2012:521), qualitative research has no direct fixed sample size; it is driven by the information needs of the study. The sample size was guided by the principle of data saturation. This point is reached when no new information is obtained from participants during the later stages of data collection, when subsequent interviews produce repetitions of information, indicating the completeness of the data gathered. In this study, data saturation was reached after interviewing 10 participants, but 2 more were interviewed to confirm that no new information could be elicited.

### Data collection

Data collection took place from September to November 2014. The researcher approached the club-goers whom she did not know personally and introduced herself and screened them based on the inclusion criteria. If the participant met the study's inclusion criteria, the researcher explained the purpose and the importance of the study. Participants were interviewed in a quiet locked room in the club to allow for privacy and minimise disruptions during interviews. Potential participants were given an information letter to read and were encouraged to ask questions to clarify matters. Consent forms were signed by those who agreed to participate. Semi-structured interviews were conducted using an interview guide. The following four questions were asked:

- Describe what made you visit the PHC clinic during the past year.
- Describe your experience at the PHC clinic starting from the time you entered the clinic, enquiries, waiting for help (in the queue), the service point (the health provider's room), waiting for medicine and receiving the medicine, leaving the clinic.
- How were you treated? Was that the way you would like to be treated? If yes, why? If not, why?
- Describe how accessible PHC services are to you.

All interviews were conducted by the researcher in English or isiZulu according to the language preference of the participant and lasted 30 to 45 minutes. All interviews were recorded using a digital recorder supplemented by field notes.

### Pre-testing of the interview guide

The interview guide was pre-tested to assess whether the research questions were realistic and understood by the pre-test participants. Pre-test participants are patients who meet the inclusion criteria but their data are not included in the actual study (Brink, Van der Walt & Van Rensburg 2012:174). The interview questions were pre-tested on two consenting participants at the clinic where the researcher worked. The data collected were not included in the final data analysis. No amendments were made to the interview questions.

### Data analysis

According to Polit and Beck (2012:556), data analysis involves organising the data that the researcher has collected, providing structure and eliciting meaning from it. Data were analysed using content analysis. Content analysis is used to analyse and interpret data by classifying the words in the text into categories in order to give it meaning. Repeated ideas or patterns of thought are grouped by organising data into categories and concepts that the researcher has developed (Grove *et al.* 2013:281).

### Ethical considerations

All consenting participants were given written and verbal information about the process of the study. They signed consent forms after reading and understanding the information letter. Privacy was maintained by not disclosing participants’ information to any third party. Justice was maintained because the participants were informed that they could decline participation at any stage of the research, or withdraw during the interview, and could do so without incurring any negative consequences. The right to confidentiality was ensured throughout the research process; names would be known only to the researcher and codes were used to identify data associated with them. The master list of participants and their codes was kept under lock and key. No family member, health worker or any other person had – or will have – access to the raw data to prevent breach of confidentiality. The Durban University of Technology Institutional Research Ethics Committee provided ethical clearance for the research proposal (IREC number 61/14).

### Trustworthiness

Guba's model of trustworthiness was applied to ensure the trustworthiness of this study in regard to credibility, dependability, confirmability and transferability (Brink *et al.* 2012:172). Credibility was maintained through using the same interview guide for all participants. The researcher visited the club over a period of three months, meeting with different participants. Member checks were conducted whereby the researcher returned to give feedback to participants and checked their reactions to themes emerging from the data. In order to ensure dependability the researcher developed an audio trail by keeping all the original audio records of interviews and discussions on a disc. To ensure confirmability, the researcher interpreted and analysed the data through identifying themes and subthemes which were supported by direct quotations from the participants in order to eliminate subjectivity and bias. A thorough description of the research report was provided to ensure transferability so that other researchers could evaluate and test the applicability of the data in other contexts.

## Results

Two major themes emerged from the data analysis:

**Theme 1:** Prejudice against homosexual patients by health care providers and other patients at PHC facilities.

**Theme 2:** Homophobic behaviour from PHC personnel.

Several sub-themes emerged from the interview in line with the two major themes. The themes and sub-themes are presented in Table 1.

**TABLE 1:** Themes and sub-themes.

Themes	Sub-themes
Prejudice against homosexual patients by health care providers and other patients at primary health care facilities.	Stigma from health care providers. Rejection by other patients. Homosexual patients' satisfaction and dissatisfaction with primary health care services.
Homophobic behaviour from primary health care personnel.	Lack of understanding of homosexuality. Attitudes of homosexual patients towards health care providers. Influence of religious and cultural beliefs of health care providers on their perceptions of homosexual patients. Heteronormativity amongst health care providers. Attitudes of health care providers towards homosexual patients. Inappropriate curiosity of health care providers regarding homosexuality. Personal involvement of health care providers with homosexual patients.

### Prejudice against homosexual patients by health care providers and other patients at primary health care facilities

Participants cited experiences of prejudice and stigmatisation as the major reasons for not receiving fair treatment from the health care providers after gaining entry to such services. Discrimination was noted by participants when they received treatment which was different from that of heterosexuals. Their perception of being stigmatised was when they were labelled on the basis of their appearance or dress code. This prejudice was experienced as coming from other patients as well as from health care providers. The sub-themes that emerged were stigma from health care providers, rejection by other patients, lack of equality for homosexual patients in PHC services, and their satisfaction and dissatisfaction with PHC services.

## Stigma from health care providers

Participants reported that they felt stigmatised by health care providers. They reported that they were being judged because of their personality and because of the way they dressed, talked or walked. Their perceptions of being stigmatised are evident in the following quotations:

‘Are you here for family planning? The nurse asked me while she was holding and reading my card. As I was getting ready for the injection she continued to say ‘you are also wearing men's underwear?’ I was there for a different injection but a nurse kept on asking about family planning, saying lesbians do not do prevention …’ (P8)

‘… [*F*]rom the nurses, umm, you get lots of questions? In the consultation room, the first question that the nurse asked me was what type of a person I was. I ignored the question and told her that I was sick …’ (P1)

‘… [*A*]t the PHC facility people have their own prejudices and there is a lot of stigmatisation. If you are a homosexual you go there looking for help for your sickness but they always include things that are unnecessary. Despite telling the nurse that you are a male and gay, they always make you to explain yourself and emphasise that you are different. They repeatedly ask if you are male or female.’ (P11)

## Rejection by other patients

Participants reported that they felt rejected by other patients in the PHC facility's waiting area. Some patients in the waiting area made comments which scared participants, causing some of them to leave the PHC facility before they were attended to by the health care practitioner. Rejection by other patients is revealed in the following quotations:

‘… [*W*]hen I went to the PHC facility, at the gate, I enquired from the security guard, an old man, who looked at me, clicked his tongue and swore at me. But I could not care less because I am used to things like that. When I got inside the PHC facility patients laughed at me because I was wearing tight pants and they were looking at my private parts, but still I did not care. I put my earphones on, ignored them and sat in the queue …’ (P7)

‘When I was still sitting in the queue there were women behind me who were laughing, but I did not know what they were laughing at, but I noticed when the woman next to me also started laughing. When I listened carefully they were debating about homosexuals. Someone said it was a fashion. The other woman added; if I can see a homosexual here I can put a tyre around her neck and burn her. I could not stay long, I decided to go back home because these people made me uncomfortable and I felt that they could harm me. I decided that when I have money I will go to the private doctor.’ (P2)

‘… I felt very disturbed and unhappy because people were sitting away from me because I am gay.’ (P5)

## Homosexual patients’ satisfaction and dissatisfaction with primary health care services

Only one participant reported satisfaction with the treatment received from the health care provider who had good communication skills and treating all patients the same. This was expressed in the following excerpt:

‘… I don't want to lie; I was so satisfied with the treatment that I got from the health care provider who was helping me. That nurse did not treat me differently from heterosexuals. She did not have that in her mind that I am a homosexual client.’ (P6)

The majority of participants reported dissatisfaction with the treatment received from health care providers. This was mainly because of the experience of participants regarding health care providers’ attitudes toward homosexual patients and of being treated differently to the way heterosexuals were being treated. This is expressed in the following quotations:

… [*I*]t is so difficult to use PHC facility. I was so dissatisfied with the treatment that I received and please do not ask how it ended. I just left the clinic without receiving any help.’ (P4)

‘… I was uncomfortable, as a result I could not disclose my real problem because I had an anal problem, I decided to go to a private doctor.’ (P7)

‘Our clinics are not in a good standard they don't accommodate all of us as we having different sexual orientations …’ (P8)

‘It was almost over in 15 minutes, a nurse busy asking me questions about being gay, and not helping me and I was no longer there I wanted help and go home but I felt very disappointed about everything.’ (P5)

## Homophobic behaviour from primary health care personnel

Participants reported that they experienced a range of homophobic behaviours from health care providers and in the waiting area from other patients. The following seven sub-themes emerged from this: lack of understanding of homosexual patients; attitudes of homosexual patients towards health care providers; influence of religious and cultural beliefs of health care providers on their perceptions of homosexual patients; heteronormativity amongst health care providers; attitudes of health care providers towards homosexual patients; inappropriate curiosity of health care providers regarding homosexuality; personal involvement of health care providers with homosexual patients.

## Lack of understanding of homosexuality

The findings of the study revealed that health care providers and members of the public who were patients in the PHC lacked an understanding of homosexuality. This was evident from the questions they asked and the disbelief that often accompanied the answers. Participants were not believed nor taken seriously when they reported that they had been infected with sexually transmitted infections, as noted in the following excerpts:

‘… [*W*]hen I got into the consultation room a nurse asked what I was suffering from and I told her that I had a problem in my private part and I think it is a sexually transmitted infection. She asked me how come I have a sexually transmitted infection while I am in a relationship with a female. The nurse asked me to explain how I have sexual intercourse. I told her that I could not explain something like that, and I asked her to help me with treatment as I wanted to leave the room.’ (P9)

‘… [*T*]he nurse asked me about my HIV status as I was having some sort of sexually transmitted infection and I told her that I was HIV negative. She asked if I was using condoms and I told her that I was not, and I told her I did not even know homosexuals’ condoms, I had never seen them before.’ (P5).

## Attitudes of homosexuals towards health care providers

Some participants who went to the PHC facility had a positive attitude towards health care providers or health facilities because of previous positive experience. One participant expressed her experience of a positive attitude regarding the PHC provider by saying:

‘The nurse who attended to me did not treat me differently. I did not feel discriminated.’ (P6)

Other participants reported that their own attitudes were negative towards the PHC providers prior to them reaching the PHC facility, because of preconceived ideas about health care providers which might or might not have been factual. This caused problems for the participants as they became agitated without provocation from the health care providers. The participants who had experienced negative attitudes from health care providers had this to say:

‘… [*W*]hat I don't like most with the clinic is the attitude of nurses towards homosexuals. I always see it …’ (P6)

‘However, I was lazy to go there because of what my friend told me, the questions he went through. Before I got helped I was asked many questions too and criticised too much.’ (P11)

’… Umm … It is not easy to use the clinic; you end up getting hurt.’ (P7)

## Influence of religious and cultural beliefs of health care providers on their perceptions of homosexuals

The religious beliefs of health care providers were perceived as problematic by participants. The participants reported that health care providers tended to want to convert or impose their religious beliefs on them and expected them to behave and lead their lives according to those beliefs. An example of such a view is expressed in the following excerpt:

‘… [*I*]t wasn't good news to a nurse to discover that I am a lesbian. The nurse started preaching saying she feels sorry for my mother, I need to go to church and pray because what I am doing is against God's will …’ (P12)

Some patients believed that homosexuality could be ‘corrected or healed’ using cultural methods they were familiar with:

‘The conversation continued until a person next to me said, being homosexual is not right, because it is Satanism and it is kind of a fashion and someone said homosexuals need slaughtering of goats because this is against culture.’ (P6)

## Heteronormativity amongst health care providers

When health care providers managed patients, they never seemed to give a thought to the fact that there could be homosexual patients seeking medical assistance. They tended to treat everyone as if they were heterosexual. Participants witnessed heteronormativity in the following instances:

‘… I told the nurse that I wanted to do an HIV test. The first question was; why do you want to do HIV test? I told her that I want to know my status; she then asked if I use condoms. Hey! I was taken aback for a moment, seriously I was confused by the question … I answered no, now a nurse gave me health education on using condoms and asked if am not aware of the importance of using condoms, I told her I knew. She did not ask me about the type of partner I have or the reason for not using condoms. Later on, she asked me if my partner has tested, and I told her that she's outside. She then asked me to call him so that she will give us both health education about the use of condoms. When my partner got inside the nurse just changed her facial expression [*frowning*] she was surprised and looked very shocked to see that it was a female. And she said: ‘this is the reason you are not using condoms’, I said ‘yes’.’ (P3)

‘… [*T*]he nurse asked the usual questions as to a female, she asked about pregnancy, my last menstrual periods, and use of condoms? When I said no to using condoms, she immediately educated me on the importance of using them. I explained my sexuality to her and she was taken aback and was speechless for some time. It wasn't good news to a nurse to discover that I'm a lesbian. Nurse started preaching me saying she feel ashamed for my mother …’ (P12)

## Attitudes of health care providers towards homosexuals

Participants reported negative attitudes from health care providers. They verbalised moments where their sexual orientation seemed to upset the health care providers. The negative attitudes of health care providers towards homosexual patients are portrayed in the following excerpts:

‘… [*W*]hen I entered to a consultation room the first thing a nurse said to me was ‘what's going on here?’, and I looked back because I thought maybe there was something happening behind me, and a nurse said no I am asking you ‘what type of a person are you really?’ (P8)

‘… [*W*]hen I got into the consultation room I explained to a nurse that I needed contraceptives, a doctor had referred me because of my abnormal menstrual periods, a nurse refused to give me contraceptives, no matter how hard I tried to explain that I am not sleeping with males I need it for abnormal periods. A nurse said I am a lesbian and I don't need contraceptives and she did not give them to me.’ (P10)

## Inappropriate curiosity of health care providers regarding homosexuality

The majority of participants reported that they had been asked about how they engaged in sexual intercourse by health care providers. The participants perceived this to be inappropriate curiosity on the part of the health care providers, and they felt that this question was irrelevant to the history taking or the diagnosis and treatment as noted in the following excerpts:

‘… [*I*]f things were done my way, I wish lesbians and gays had a dedicated clinic in order not to get tortured by nurses with lots of questions, that are private like asking how do we engage in sexual intercourse with the person of the same sex?’ (P12)

‘… [*T*]he question that followed was; how do you engage in sexual intercourse as you're both females?’ (P8)

‘… [*T*]he nurse asked me to explain how I have sexual intercourse. I told her that I could not explain something like that, and I asked her to please help me with treatment as I wanted to leave the room.’ (P9)

## Personal involvement of health care providers with homosexuals

Sometimes health care providers were somewhat personally affected by homosexuality either having a homosexual child or the health care provider admitting to having feelings for homosexual patients. Some participants felt offended whilst others felt that this helped the health care provider understand homosexuality better. An example of a participant who felt offended is provided below:

‘… [*W*]hen I got inside the consulting room the male nurse asked if he should write female or male, I told him that I am a gay male, I am not a female. After that he told me that he was married but wished to have relations with a gay person like me. I became so angry because I thought that he was provoking me. He then told me that we will book a place where we can sleep. I was so annoyed and uncomfortable as he was continuing with the conversation …‘ (P7)

## Discussion

### Prejudice against homosexual patients by health care providers and other patients at primary health care facilities

Participants identified prejudice against them as an obstacle to their utilisation of health care services. This is consistent with findings of other studies (for example Kennedy *et al.* 2013; Müller 2013; Rounds *et al.* 2013). Rosenstreich *et al.* (2011:302) state that throughout the world health care providers and communities still discriminate against and marginalise homosexual patients. Some health care providers lack the tolerance required to work with them because of their personal affiliations which interfere with their professionalism (Röndahl 2009:146). Many health care providers found it difficult to render care to them as they cannot tolerate their sexuality and end up failing to give quality nursing care equal to that given to heterosexuals (Röndahl 2009:147). Participants in this study reported that they were not treated in the same way as heterosexuals. Only one participant reported the experience of being treated fairly. Two studies report that patients verbalised satisfaction with health care services when their health needs were met (McNair, Szelacha & Hughes 2011; Tjepkema 2008). These authors further assert that satisfaction is reported to be higher when health care providers show acceptance and respect towards patients’ homosexuality and display positive attitudes towards them. Rounds *et al.* (2013:99) reported that homosexual patients received substandard care from health care providers.

Participants verbalised that some members of the public who were patients showed signs of prejudice by rejecting homosexual patients in the waiting areas at the clinics where all patients queue whilst waiting for assistance from the health care providers. Some reported leaving the PHC facilities before consultation with the health care providers because of this. According to Araújo *et al.* (2009:667), homosexual patients usually experience situations of prejudice in the community. The burden of stigma might be more severe for patients who look like homosexual patients compared to those who are not easily identifiable. These authors further state that homosexual patients might be humiliated and mocked by some members of society even in the health care settings because of their homosexuality.

### Homophobic behaviour from primary health care personnel

The participants in the current study reported experiencing homophobic behaviour from PHC personnel during their utilisation of health care services. According to Akhan and Barlas (2013:435) homophobia lies behind the judgements, negative attitudes and unfair treatment of homosexual patients in health care facilities. Berry *et al.* (2013:40) state that members of the public are generally not educated about, and lack an understanding, of homosexual people and once they discover that someone is homosexual, they change their attitudes and start looking down upon them.

Participants reported that there were moments where the health care providers preached to them instead of providing the services that they required. Röndahl (2009:149) argues that the religious health care providers can be problematic in the nursing context, especially when nursing homosexual patients. They became more concerned about influencing homosexual patients to become heterosexuals instead of treating patients holistically. This attitude can also be present in married health care providers who associate homosexuality as being against God's will for what a family should be and therefore it needs to be changed (Akhan & Barlas 2013:435). Some participants in this study experienced criticism from members of the public who were also patients in the PHC, and who believed that homosexuality was against the cultural background of African people. According to Akhan and Barlas (2013:435), these homophobic attitudes are influenced by the social arrangements and the patriarchal structures of countries. An example of patriarchal heteronormative behaviour was experienced by participants in the current study when it was assumed that they were heterosexual because health care providers did not ask about their sexual behaviour. Lesbians are becoming more visible in society but health care providers still tend to treat all females as if they are heterosexuals (Hayman *et al.* 2013:121).

One participant in the current study verbalised that she had a positive meeting with the health care provider who showed good communication skills and acknowledgement of homosexuality. According to Röndahl (2009:146), the insecurities of homosexual patients sometimes affect their interaction with health care providers as not all health care providers display negative attitudes towards them.

In this study some health care providers indicated inappropriate curiosity when they constantly enquired about how homosexuals engage in sexual relations. Hayman *et al.* (2013:120) report cases of health care providers asking about how conception takes place, laughing and joking about pregnancies amongst homosexual women. However, health care providers sometimes ask homosexual patients about their sexuality and sexual activities in order to understand their personal issues. Akhan and Barlas (2013:435) suggest that when health care providers meet homosexual patients they should seize the opportunity to educate themselves and their families about their lifestyle.

## Limitations of the study

Researching homosexual patients in South Africa is challenging because they are hard to access as many have not ‘come out’ about their homosexuality, especially homosexual males; hence, only those who visited a particular gay club in Umlazi were interviewed. The research was conducted on those residing in Umlazi only. The experiences of health care providers were not studied, only that of homosexual patients. Participants were not asked about their experiences of accessing and utilising private health care services, as this aspect fell beyond the scope of the current study.

## Recommendations

The following recommendations emerged:

- Nursing education institutions, in collaboration with the National Department of Health, should introduce homosexuality and anti-homophobia education programmes during the pre-service and in-service education period. Such programmes will help to familiarise health care providers with the health care needs of homosexual patients and may decrease homophobic attitudes.
- Guidelines on history taking from homosexual patients should be compiled. This will improve their ability to offer relevant health information and management.
- Health care providers should not show patients that they are surprised about their sexual orientation. They need to set a relaxed tone and be aware of their negative body language.
- Campaigns to educate the public about homosexuality could create a more conducive environment for homosexual patients so that they can access health facilities without fear of prejudice and discrimination by members of the public who are also patients at the PHCs. Such education sessions could be provided in PHC clinic waiting areas and during HIV and/or AIDS health education sessions.
- The possibility should be investigated of offering PHC services at gay clubs, for instance one evening per week, by health care providers who are willing to treat homosexual patients with respect and without prejudice.
- Future studies should endeavour to identify the attitudes of health care providers working at PHC clinics towards homosexual patients, as well as their knowledge of and perceptions of them.

## Conclusion

The findings indicate that some participants encountered challenges to accessing and using PHC services in the Umlazi area. The major patient-related challenge was that some participants feared discrimination at the PHC clinics and consequently they postponed or abandoned their PHC clinic visits. The major service-related challenges involved the non-professional attitudes of some health care providers who discriminated against some participants’ sexual orientations, asked inappropriate questions and failed to provide adequate services. Some of the other patients in the waiting areas also showed disrespect towards participants causing some of them to fear for their safety to the extent that they left the clinic without consulting any health care professional.

Health care providers should receive pre-service and in-service education about the necessity of treating homosexual patients professionally and respectfully. Members of the public should also be educated about homosexuality and about avoiding discriminating against homosexual patients. Such education sessions could be provided in PHC clinic waiting areas, at women's and men's gatherings and during HIV and/or AIDS health education sessions. Implementation of some of these recommendations could help to facilitate homosexual patients access to and utilisation of PHC services in the Umlazi area of the KwaZulu-Natal province.
